# Inter-center comparison of good manufacturing practices-compliant stromal vascular fraction and proposal for release acceptance criteria: a review of 364 productions

**DOI:** 10.1186/s13287-021-02445-z

**Published:** 2021-07-01

**Authors:** Pauline François, Giulio Rusconi, Laurent Arnaud, Luca Mariotta, Laurent Giraudo, Greta Minonzio, Julie Veran, Baptiste Bertrand, Chloé Dumoulin, Fanny Grimaud, Luc Lyonnet, Dominique Casanova, Camille Giverne, Audrey Cras, Guy Magalon, Françoise Dignat-George, Florence Sabatier, Jeremy Magalon, Gianni Soldati

**Affiliations:** 1 Cell Therapy Department, Hôpital de la Conception, AP-HM, INSERM CIC BT 1409, 147 Bd Baille, 13005 Marseille, France; 2 Aix Marseille Univ, INSERM, INRA, C2VN, Marseille, France; 3 Swiss Stem Cell Foundation, Gentilino, Lugano, Switzerland; 4 Department of Biotechnology and Life Sciences, University of Insubria, Varese, Italy; 5 Vascular Biology Department, Hôpital de la Conception, AP-HM, Marseille, France; 6 Plastic Surgery Department, Hôpital de la Conception, AP-HM, Marseille, France; 7 Normandie Univ, UNIROUEN, INSERM, U1234, Rouen University Hospital, Department of Immunology and Biotherapy, Rouen, France; 8 Assistance Publique-Hôpitaux de Paris, Saint-Louis Hospital, Cell Therapy Unit, Cord blood Bank and CIC-BT501, Paris, France; 9 Remedex, Marseille, France

**Keywords:** Stromal vascular fraction, Adipose tissue, Cell subset distribution, Flow cytometry, GMP production, Advanced therapy medicinal product

## Abstract

**Background:**

Even though the manufacturing processes of the stromal vascular fraction for clinical use are performed in compliance with the good manufacturing practices applying to advanced therapy medicinal products, specifications related to stromal vascular fraction quality remain poorly defined. We analyzed stromal vascular fraction clinical batches from two independent good manufacturing practices-compliant manufacturing facilities, the Swiss Stem Cell Foundation (SSCF) and Marseille University Hospitals (AP-HM), with the goal of defining appropriate and harmonized release acceptance criteria.

**Methods:**

This retrospective analysis reviewed the biological characteristics of 364 batches of clinical-grade stromal vascular fraction. Collected data included cell viability, recovery yield, cell subset distribution of stromal vascular fraction, and microbiological quality.

**Results:**

Stromal vascular fraction from SSCF cohort demonstrated a higher viability (89.33% ± 4.30%) and recovery yield (2.54 × 10^5^ ± 1.22 × 10^5^ viable nucleated cells (VNCs) per mL of adipose tissue) than stromal vascular fraction from AP-HM (84.20% ± 5.96% and 2.25 × 10^5^ ± 1.11 × 10^5^ VNCs per mL). AP-HM batches were significantly less contaminated (95.71% of sterile batches *versus* 74.15% for SSCF batches). The cell subset distribution was significantly different (higher proportion of endothelial cells and lower proportion of leukocytes and pericytes in SSCF cohort).

**Conclusions:**

Both centers agreed that a good manufacturing practices-compliant stromal vascular fraction batch should exert a viability equal or superior to 80%, a minimum recovery yield of 1.50 × 10^5^ VNCs per mL of adipose tissue, a proportion of adipose-derived stromal cells at least equal to 20%, and a proportion of leukocytes under 50%. In addition, a multiparameter gating strategy for stromal vascular fraction analysis is proposed.

**Supplementary Information:**

The online version contains supplementary material available at 10.1186/s13287-021-02445-z.

## Background

The stromal vascular fraction (SVF) is an heterogeneous cell population extracted from adipose tissue (AT) [1–3]. For several years, it has been classified as an advanced therapy medicinal product (ATMP) and evaluated in multiple clinical trials for its regenerative ability. Indeed, initially formalized by Bourin et al., the SVF contains at least 15% of mesenchymal stromal/stem cells (MSCs) also called adipose-derived stromal cells (ASCs), 10 to 20% of endothelial cells and progenitors (ECs), and about 4% of pericytes (PRs). These different cell subsets cooperate to promote synergistic angiogenic, immunomodulatory, and trophic effects [4]. SVF also contains blood cells and notably leucocytes (Leuk) although their exact contribution to the biological effect of SVF is not elucidated.

As an ATMP, the SVF must be produced in compliance with the good manufacturing practices (GMP) and thus necessitates quality controls during production, mainly in order to ensure cleanliness of the production area and inherent sterility of the final product. The viability of the cell product is always determined as it conditions cell integrity and function and is essential to ensure a potential therapeutic effect. In this context, SVF viability is often considered as the main specification of the final ATMP. As well as the viability, the cell recovery rate or cell yield corresponding to the number of viable nucleated cells (VNCs) obtained per mL of AT is usually estimated and is a good indicator of the extraction efficiency. Indeed, it is now well established that technical settings associated to AT processing can influence SVF characteristics and efficacy. They include both conditions of AT harvesting such as the harvesting area [5], the harvesting technique [6, 7], and conditions of manufacturing [8, 9] according to whether they are manual or automated, enzymatic, or mechanic [10–14]. Furthermore, flow cytometry is classically used to define the distribution of the main nucleated cell subsets within isolated SVF, as the best approach to approximate the SVF composition. Because of the diversity of potential mechanisms of action associated to in vivo delivery of SVF, and the difficulty to anticipate them through in vitro testing, its cellular composition is currently viewed as surrogate marker of SVF potency. However, flow cytometry analysis of clinical grade SVF and results’ interpretation is crucially limited by a lack of standardization. Although guidelines are available and indicate which markers should be used to identify each SVF cell population, no consensus emerges when looking at a large body of articles [15–20]. The SVF composition is highly different according to studies and no reference values exist to qualify SVF prior to clinical use. This issue also compromises the identification of a potential relationship between SVF composition and therapeutic activity.

In an attempt to contribute to a better harmonization and standardization of practices in the field of SVF-based therapy, we conducted a retrospective analysis of a large number of clinical-grade SVF batches released by two nationally authorized GMP-compliant cell therapy facilities: the Swiss Stem Cell Foundation (SSCF) in Switzerland and the cell therapy department of Marseille University Hospital (AP-HM) in France. The objective was to compare the manufacturing processes and SVF biological attributes resulting from quality control testing in order to define a control strategy and delineate relevant thresholds for release acceptance criteria.

## Methods

### Donor specifications

AT was obtained from two cohorts of patients included either in the service offered by SSCF, Switzerland, for aesthetic purpose or in the French clinical trials listed in supplemental table 1. For the latter, AT harvesting was performed in the department of plastic surgery, La Conception, University Hospital, Marseille, France. The study was conducted in accordance with the Declaration of Helsinki, and all subjects provided informed consent. If necessary, studies were approved by national ethics committee review board (supplemental table 1) between January 2013 and middle 2019.

### Adipose tissue harvesting

For the SSCF cohort, AT harvesting was performed in an operating room under general anesthesia. AT was collected in three 60-ml syringes filled with a maximum of 50 ml each with various cannula; syringes were inserted in a double secondary container (2 labeled envelopes), then delivered to SSCF in a dedicated transport container at room temperature (20 ± 10 °C). The AT sample was processed within 24 h after collection.

For the AP-HM cohort, AT harvesting was performed in an operating room under general or local anesthesia after a standardized skin aseptic preparation using a Khouri cannula (Khouri Harvester, Koume, Lipoplasty Products, Sunrise, FL, USA), a 12-gauge 12-hole multiperforated cannula, through a closed circuit, preventing contamination of the harvested product. Then, the AT was packaged in a bag (Easyflex+, Macopharma, Mouvaux, France). Once harvesting was complete, the bag was transported to the cell therapy unit and the AT was immediately processed.

### SVF manufacturing

In the SSCF facilities, AT was washed twice with Dulbecco’s phosphate-buffered saline with calcium and magnesium solution (DPBS +/+, Gibco, Life Technologies, Carlsbad, CA, USA) and enzymatically digested for 45 min at 37 °C under constant agitation in a cell culture incubator. For the latter, Liberase® MNP-S (Roche, Basel, Switzerland), i.e., a mix of collagenase I and II, and neutral proteases, was used. Enzymatic digestion was then stopped with sterile, clinical grade 1% human serum albumin (HSA, CSL Behring, King of Prussia, PA, USA) in DPBS without calcium and magnesium (DPBS −/−, Life Technologies); the hydrophilic phase was collected and subsequently filtered with 100-μm and 40-μm sieves. At the final step of SVF manufacturing, the hydrophilic phase was centrifuged and the resulting cell pellet, i.e., the autologous SVF, was resuspended in 5% HSA.

In the AP-HM facilities, the autologous SVF was obtained using the Celution 800/CRS automated processing system (Lorem Cytori, San Diego, CA, USA) according to the manufacturer’s instructions. Briefly, the collected lipoaspirate was washed with Ringer’s lactate (RL; Baxter, Deerfield, IL, USA) and enzymatically digested with Celase, a GMP cocktail of enzymes provided with consumables (Worthington Biochemical Corp., Lakewood, NJ, USA). The cells were concentrated, washed, aseptically recovered, and resuspended in RL.

### Viable nucleated cell concentration and cell viability

For both cohorts, the number of VNCs and percentage of cell viability were determined using a NucleoCounter NC-100 instrument (ChemoMetec, Allerød, Denmark). Recovery yield was calculated as the number of total VNCs obtained divided by the initial volume of AT measured after removal of infiltration liquid (decantation).

### Microbiological testing and environmental monitoring

For both cohorts, a microbiological testing was performed on the end product consisting in inoculating SVF samples in Bact/Alert culture bottles (aerobic and anaerobic culture vials, each containing 40 mL of medium). The Bact/Alert method (Biomerieux, Marcy l’Etoile, France) uses a computer-controlled incubation/detection system. The media used contained proprietary factors designed to inactivate a wide variety of antibacterial and antifungal agents. Bact/Alert culture bottles were incubated at 37 °C and 5% CO_2_ for a total of 10 days (AP-HM) or 14 days (SSCF), and automated readings were taken every 10 min (according to the *European Pharmacopoeia - Chapter 2.6.27 Microbiological Examination of cell-based Preparations*). Detection of organisms resulted in an audible alarm and automatic recording of time of detection.

Some “in process” environmental monitoring quality controls were also carried out in both facilities, consisting in contact agar, gloves fingerprints, or air impacting. The environmental controls details and a summary of both protocols are exposed in Table 1.

**Table 1:** Comparative description of SSCF and AP-HM methods. Comparative description of each critical steps of SVF manufacturing and associated quality controls performed at the SSCF and AP-HM cell-therapy facilities. *DPBS* Dulbecco’s phosphate-buffered saline, *HSA* human serum albumin, *RL* Ringer’s lactate

	SSCF	AP-HM	
**Adipose tissue harvesting**	Manual or automatic harvesting using various canula	Manual harvesting using a Khouri Cannula after a strict aseptic skin preparation	
**Settling**	10 min	5 min	
**Packaging in syringes**	Directly packaging in 60-mL Luer Lock syringes.	Packaging in 60-mL Luer Lock syringes from a bag	
**Device preparation**	Manual collection of AT	Tensioning the Celution® device, settlement of consumables, and seal check	
**Adipose tissue transfer**	Into three 100-ml syringes	AT transfer in the Celution® device	
**Settling**	10 min	Automated	Visual check of discarding infiltration liquid
**Infiltration liquid removal**	Yes		
**Weighing**	150 ml of AT required		At least 100 mL of AT required
**Washing**	Twice with DPBS +/+		From two to five washings with RL and visual check
**Addition of the enzyme**	Addition of Liberase®	Addition of Celase® in the device	
**Adipose tissue digestion**	45 min at 37 °C	Automated	20 min at room temperature
**Washing**	DPBS −/− + 1% HSA	Automated	With RL
**Filtration**	100 μm and 40 μm	NA	
**Collection of the SVF**	SVF resuspended in 5% HSA	SVF resuspended in RL	
**Quality control sampling**	1) Cell count and viability 2) Evaluation of cell subset distribution with flow cytometry	1) Cell count and viability 2) Evaluation of cell subset distribution with flow cytometry 3) CFU-F assay	
**Quality control of the end product**	Microbiological control	Microbiological control	
**Environnemental control in process**	1) Passive agar (A class) (2x) 2) Contact agar bench (2x) 3) Gloves fingerprints after SVF packaging (2x) 4) Air impacting (A and B class)	1) Passive agar (B class) 2) Passive agar (A class) 3) Gloves fingerprints after AT transfer 4) Gloves fingerprints after enzyme reconstitution and transfer 5) Gloves fingerprints after SVF collection 6) Contact agar bench 7) Contact agar working area 8) Gloves fingerprints after SVF packaging 9) Contact agar working area 10) Air impacting (A and B class)	

### Flow cytometry analysis of SVF cell subsets

#### Immunolabeling of SVF cells

For SSCF cohort, the SVF cells were characterized by cytofluorimetric analysis using a 10 channel Navios cytometer (Beckman Coulter, Nyon, Switzerland). Briefly, 500,000 VNCs were sampled and centrifuged 5 min at 400*g*. The pellet was resuspended in 220 μL of DPBS without calcium and magnesium (Life Technologies) supplemented with 1% human serum off the clot (PAA Laboratories, Inc); then, 100 μL of the cell suspension was distributed into two test tubes and stained with the Syto40 nuclear marker, the 7AAD viability marker and antibodies or corresponding isotype controls in matched concentrations. The monoclonal antibody mix, ready to use and lyophilized (Beckman Coulter), included antibodies directed against CD146, CD45, and CD34, conjugated with the following fluorochromes: PE, KRO, and APCA750 respectively (references listed in supplemental table 2). After 20 min of incubation, erythrocytes were lysed with 1 mL of VersaLyse (Beckman Coulter). Before acquisition, 100 μL of Perfect-Count Microspheres (Cytognos, Salamanca, Spain) was added to the test tube. Data files were analyzed using Kaluza software (Beckman Coulter).

For AP-HM cohort, characterization of the SVF cell sub-populations was performed by flow cytometry using also a 10 channel Navios instrument (Beckman Coulter, Brea, CA, USA). Aliquots of 500,000 VNCs per tube were resuspended in 100 μL of DPBS, after centrifugation 5 min at 400*g*, and stained for 20 min at room temperature in the dark with the DRAQ5 nuclear marker and ready to use antibody mixes or corresponding isotype controls in matched concentrations. The monoclonal antibodies mix included antibodies against CD90, CD146, CD34, and CD45, conjugated with the following fluorochromes: FITC, PE, ECD, and PC5 respectively (references listed in supplemental table 2). Red blood cells were lysed in NH_4_Cl for 10 min before the cells were centrifuged and resuspended in DPBS without calcium and magnesium (Life Technologies). Then, NucBlue (Thermo Fisher Scientific), which allows discrimination of viable and dead cells, was added for 5 min prior to flow cytometry analysis. Data files were analyzed using Kaluza software (Beckman Coulter).

#### Inter-center validation of a common flow cytometry gating strategy

**Fig. 1:**
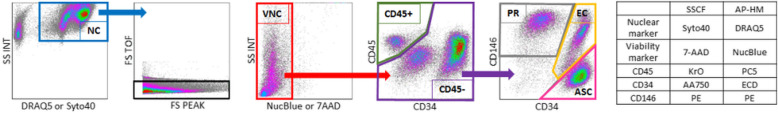
The common gating strategy. Presentation of the common gating strategy and reminder of the fluorochromes conjugated according to the initial protocol of each center. EC, endothelial cell; ASC, adipose-derived stromal cell; PR, pericyte; SS, size scatter; FS, forward scatter; NC, nucleated cell; VNC, viable nucleated cell

In order to be able to reliably compare the flow cytometry data generated by each center, we first verified that the difference in the antibodies panel used by the two facilities did not hamper the comparability of data when a common gating strategy is used. To that aim, 14 SVF batches manufactured in the two centers using a similar manual digestion protocol [21] were immunolabeled using the SSCF antibodies panel and the AP-HM antibodies panel. Samples were then analyzed by the same operator using the same defined gating strategy using Kaluza software as follows (Fig. 1):

##### Common gating strategy

1. Nucleated cells (NCs) were selected according to the nuclear marker used (DRAQ5 or Syto40).
2. Aggregates were removed using the forward scatter.
3. Viable nucleated cells (VNCs) were determined based on the death marker (NucBlue or 7-AAD).
4. Among VNCs, the CD45 marker was used to discriminate the hematopoietic CD45+ cells from the non-hematopoietic regenerative cells CD45−.
5. A density plot CD146 *versus* CD34, gated on the CD45− cells, allowed the identification of ASCs (CD45-CD34+CD146-), EC (CD45-CD34+CD146+), and an heterogeneous populations containing PRs and transitional cells (CD45-CD34-CD146+).

That strategy leads to a complete characterization of the SVF, meaning the addition of the proportions of ASCs, ECs, PRs, and transitional cells and hematopoietic cells equals to 100%.

### Statistical analysis

Statistical analysis were performed using Graph Pad Prism 5 (GraphPad Software, La Jolla, CA, USA). Quantitative variables are reported as the mean ± standard deviation (SD). The nonparametric Mann–Whitney t-test was used for the cohorts comparison. Paired t-test was used for the validation of the common gating strategy. A *p*-value < 0.05 was considered to indicate a statistically significant difference. In addition, to establish the release acceptance criteria, we have chosen the best thresholds achievable in at least 80% of cases for the parameters which are related to potency.

## Results

### Characteristics of donors and AT harvesting

A total of 364 SVF from 294 patients included in the SSCF cohort and 70 patients included in the AP-HM cohort were available for analysis. Characteristics of donors are summarized in Table 2. AP-HM cohort was composed of a greater proportion of female (*p* < 0.0001) and was slightly older compared to SSCF cohort (*p* = 0.0570). In AP-HM cohort, only manual AT harvesting was performed whereas in SSCF cohort, the manual harvesting method was used in only 66.30% of cases. In SSCF cohort, surgeons are free to choose the canula, whereas in AP-HM cohort, only Khouri canula is used. The harvesting area distribution were similar between both cohorts (*p* = 0.1138). The volume of harvested AT was significantly higher (*p* < 0.0001), but less variable in AP-HM cohort than in SSCF cohort with a coefficient of variation of 27.83% versus 41.85% and volumes ranging from 100.00 to 340.00 mL and from 20.00 to 450.00 mL in AP-HM and SSCF cohorts respectively.

**Table 2:** Baseline characteristics of patients. Data are presented as mean ± SD, or as percentages

	SSCF, ***n*** = 294	AP-HM, ***n*** = 70	***p***-value
**Gender, female/male**	60.9%/39.1%	88.6%/11.4%	< 0.0001
**Age, years, mean ± SD**	49.23 ± 10.02	51.51 ± 13.77	0.0566
**Volume of the harvested adipose tissue (without the infiltration liquid)**	130.1 ± 54.45 mL	201.9 ± 56.18 mL	< 0.0001
**Harvesting methods**			
**Automatic aspiration**	18.0%	-	-
**Manual lipoaspiration (Khouri canula)**	-	100.0%	
**Manual lipoaspiration (various canula)**	66.3%	-	
**Method for lipoaspiration unknown**	15.6%	-	
**Harvesting area**			
**Abdomen**	37.1%	31.4%	0.1138
**Multiple sites**	26.5%	22.9%	
**Unknown**	17.3%	17.1%	
**Flanks**	9.9%	5.7%	
**Others (back, knee, arm and thighs)**	9.2%	22.9%	

### Viability and recovery yield

**Fig. 2:**
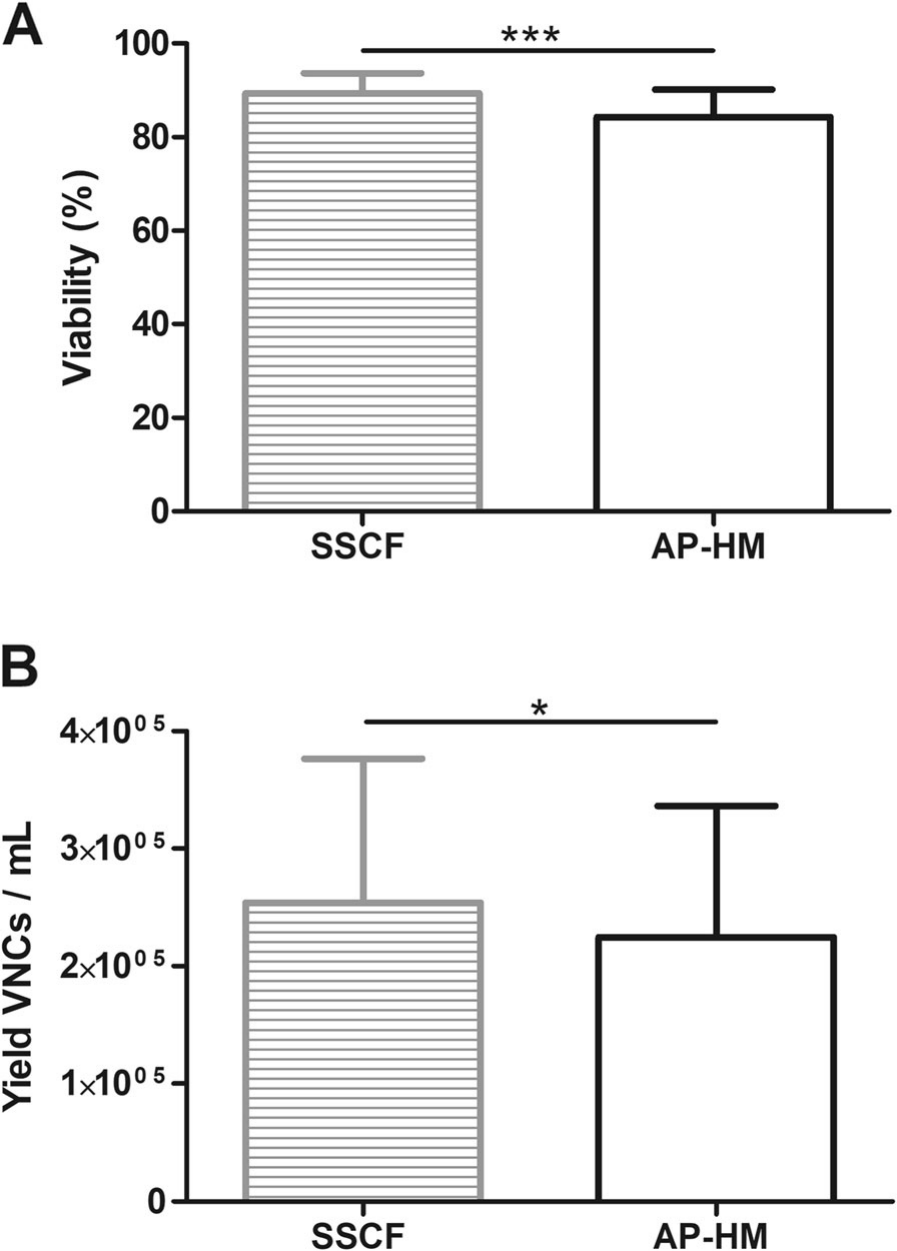
Comparatives analyses of viability percentage and yield recovery of VNCs/mL AT between the two cohorts. Viability of SVF cells was higher in SSCF cohort compared to AP-HM cohort (*p* < 0.0001). Yield of VNCs/mL of AT was higher in SSCF cohort (*p* = 0.041). VNC, viable nucleated cell; SSCF, Swiss Stem Cell Foundation; AP-HM, *Assistance Publique-Hôpitaux de Marseille*; AT, adipose tissue

SVF viability was higher in SSCF cohort with a mean percentage of 89.33% ± 4.30% compared to the AP-HM cohort (84.20% ± 5.96%, *p* < 0.0001) (Fig. 2A). Recovery yield was also higher in SSCF cohort with an average of 2.54 × 10^5^ ± 1.22 × 10^5^ VNCs extracted per mL of AT compared to 2.25 × 10^5^ ± 1.11 × 10^5^ VNCs recovered in AP-HM cohort (*p* = 0.0408) (Fig. 2B).

### Microbiological controls and environmental monitoring

**Fig. 3:**
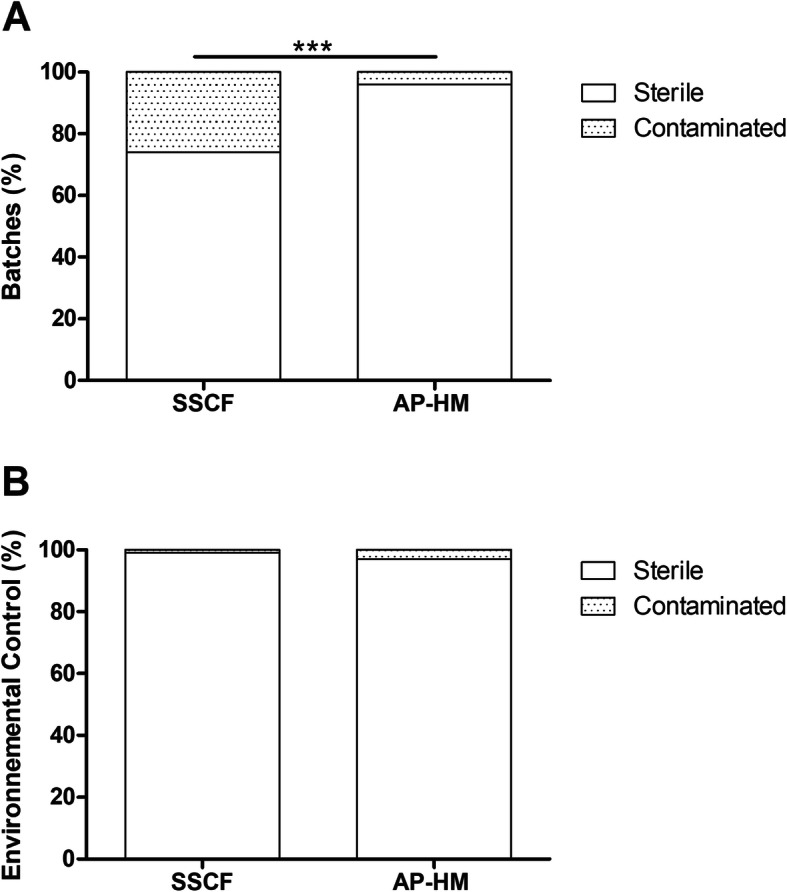
Free of germs and contaminated batches of the finished product and environmental monitoring within the two cohorts. **A** AP-HM batches were significantly less contaminated compared to SSCF batches (*p* = 0.0008). **B** No significant differences were observed for environmental monitoring, data missing for 29 patients in SSCF cohort and 12 patients in AP-HM cohort; SSCF, Swiss Stem Cell Foundation; AP-HM, Assistance Publique-Hôpitaux de Marseille

AP-HM batches were significantly less contaminated with 95.71 % of sterile batches *versus* 74.15% of sterile batches in SSCF cohort (*p* < 0.0001) (Fig. 3A). Regarding in process environmental monitoring, no significant difference could be evidenced between the two manufacturing facilities: 99.80% (1852 out of 1855) of controls performed at SSCF, and 97.10% (563 out of 580) of controls performed at AP-HM were found to be free of germs (*p* = 0.6212) (Fig. 3B).

### Validation of flow cytometry common gating strategy

**Fig. 4:**
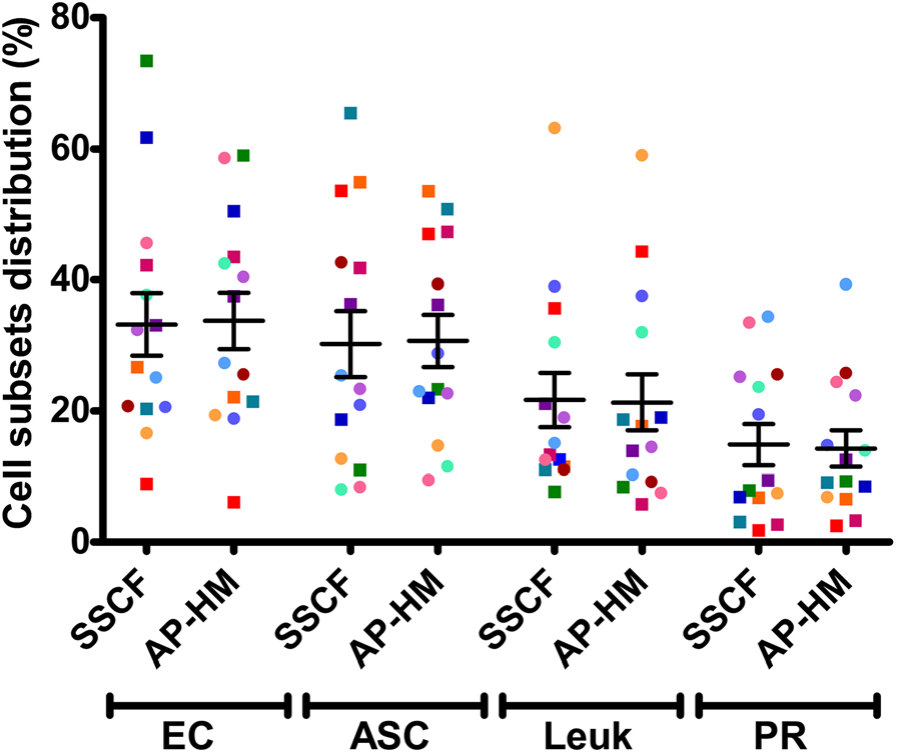
Validation of the common gating strategy. A multicenter analysis was performed to validate the common gating strategy. Fourteen AT samples were analyzed by flow cytometry with the two antibody panels, seven in AP-HM facilities and seven in SSCF facilities. The analysis was performed by the same operator using the common gating strategy. Each color represents a batch. Squares represent experiments realized in SSCF facilities. Rounds represent experiments realized in AP-HM facilities. No difference could be observed in cell subset distribution. EC, endothelial cell; ASC, adipose-derived stromal cell; Leuk, leukocytes; PR, pericyte

A multicenter study was made for validation of flow cytometry experiments. Fourteen SVF batches manufactured using similar manual enzymatic digestion were analyzed using the commonly defined gating strategy and comparing the different antibodies mixes used in the two centers SSCF and AP-HM. In these conditions, no significant difference could be noted between cell subsets distribution as evidenced by the proportions of ECs (*p* = 0.7819), ASCs (*p* = 0.7947), leukocytes (*p* = 0.8014), and PRs (*p* = 0.6290) determined in the two facilities. (Fig. 4). This result indicates that when the common strategy is used, results from flow cytometry analysis of the SSCF and APHM cohorts can be reliably compared in retrospective analysis.

### Cell subset distribution

**Fig. 5:**
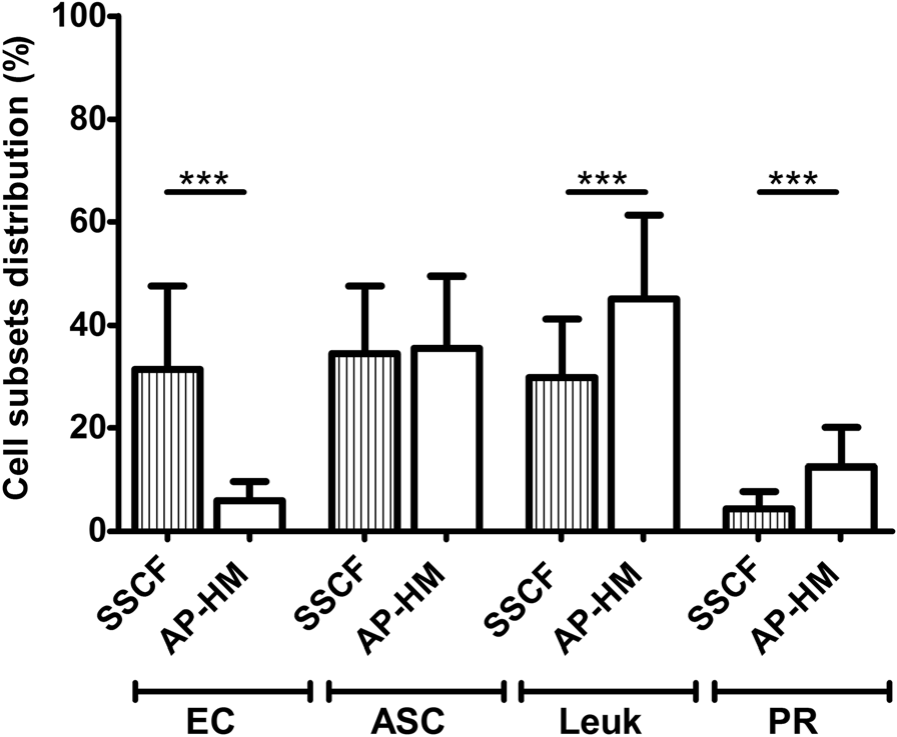
Comparison between the cell subset distribution of the two cohorts. Phenotypic data were reanalyzed using the common gating strategy. EC were found to be in a greater proportion in SSCF cohort (*p* < 0.0001). No significant difference could be evidenced for the ASCs distribution (*p* = 0.5559). Proportion of leukocytes was higher in AP-HM cohort (*p* < 0.0001). Proportion of PR was also higher in AP-HM cohort (*p* < 0.0001). EC, endothelial cell; ASC, adipose-derived stromal cell; Leuk, leukocytes; PR, pericyte

Flow cytometry results indicate that the proportion of ECs was significantly higher in SSCF cohort compared to AP-HM cohort (31.34% ± 16.34% *versus* 5.94% ± 3.71% respectively; *p* < 0.0001). However, no significant difference was observed for ASCs distribution (34.53% ± 13.09% *versus* 35.52% ± 13.96%; *p* = 0.5559). Finally, leukocytes and PRs were found in a lower proportion in SVF from SSCF cohort with respective percentages of 29.81% ± 11.41% and 4.31% ± 3.37% *versus* 45.17% ± 16.17% and 12.51% ± 7.67% for AP-HM cohort (*p* < 0.0001 and *p* < 0.0001 respectively) (Fig. 5).

### Proposal for release acceptance criteria thresholds

In the light of these retrospective data collected by SSCF and AP-HM centers from a large number of therapeutic-grade SVF batches, some essential criteria can be put forward to standardize the release of SVF batches manufactured in compliance with GMP guidelines.

1. Viability of fresh SVF assessed by a cell counter should be equal or superior to 80%.
2. The recovery yield after extraction should be a minimum of 1.50 × 10^5^ VNCs per mL of total harvested AT, after removal of the anesthetic infiltration liquid.
3. Proportion of ASCs in the end product should be at least equal to 20%.
4. Proportion of leukocytes in the end product should be less than 50%.
5. Microbiological testing should evidence the sterility of SVF product.

Among the 364 batches of the GMP-SVF analyzed, the viability threshold was reached for 92.90% of them (representing 338 batches); the recovery yield threshold was acquired for 81.30% of them (representing 296 batches); 86.80% of the SVF presented a proportion of ASCs equal or superior to 20% (representing 316 batches); finally, 87.40% of the batches demonstrated a proportion of leukocytes under 50.00% (representing 318 productions). Any center combined, the overall proportion of SVF batches reaching the microbiological attribute is 96.94%.

## Discussion

As far as we know, this study is the first to provide a retrospective analysis of the quality attributes of GMP compliant SVF administered to patients. It takes place in the context of the increasing popularity gained by SVF as an experimental ATMP and its evaluation in many fields of regenerative medicine supported by positive outcomes from early-stage clinical trials. Nonetheless, the heterogeneity of this cell-based product associated to other variability factors such as donor characteristics, manufacturing procedures, or analytical methods have led to a consensus view recommending increased harmonization of quality control and batch release criteria. As specified in appendix B, “considerations for development of final product release criteria specifications and stability protocols” of the guidance for FDA reviewers and sponsors (“Content and Review of Chemistry, Manufacturing, and Control (CMC) Information for Human Somatic Cell Therapy Investigational New Drug Applications (INDs)”) [22], the proposed release acceptance criteria should be based on scientific data and manufacturing experience. This referral only mentioned an expected viability rate greater than 70% for any cell-based product. Former recommendations dedicated to adipose tissue-derived products were provided in 2013 by the International Federation for Adipose Therapeutics and Science (IFATS) and the International Society for Cellular Therapy (ISCT), but mainly concerned the adipose mesenchymal/stromal cells. In addition, they have never been matched to numerous data from real-life use of therapeutic SVF.

Our observation first indicated that the viability and recovery yield of SVF were higher in SSCF cohort when a manual extraction protocol was used. Indeed, the enzymatic digestion automatically performed by the Celution device is shorter than the SSCF digestion and is happening at room temperature whereas the optimal temperature for enzyme activity is 37 °C [23]. Since 2020, the AP-HM has changed its SVF production protocol for a more efficient manual process [21]. Furthermore, the majority of patients (sixty-two patients) included in the AP-HM cohort presented a chronic inflammatory disease such as systemic scleroderma or Crohn disease; the SVF may be impacted by this inflammatory signature and that could also explain the lower viability and recovery yield observed in the AP-HM cohort. The size and the shape of adipose tissue harvesting cannulas used are known to influence the biological characteristics of the final SVF and could partly explained the observed differences between SSCF and AP-HM protocols [5, 6, 24].

Conversely, SVF from AP-HM were significantly less frequently contaminated with 95.71% of aseptic batches. Although poor data on the microbiological contamination of SVF-based cell therapy products are available, this result is probably explained by a stricter and standardized protocol of skin asepsis before adipose tissue harvesting, implemented according to recommendations from the nosocomial infection control committee from AP-HM. Indeed, AP-HM use a skin decontamination protocol in seven times: application of Betadine scrub (Mylan, Canonsburg, Pa, USA), rinsing with a sterile saline solution, time of drying, application of Betadine scrub again, rinsing with a sterile saline solution, time of drying, and finally application of alcoholic Betadine (Mylan). In addition, patients have two preoperative showers. Furthermore, the AP-HM team carried out ten environmental microbiological monitoring during SVF manufacturing whereas SSCF team performed only four. To date, there is no consensus on the sampling plan (number and nature) of the environmental controls to be performed and each producer should justify the testing strategy based on a risk analysis. The use of the Celution 800/CRS device requires manipulation in a microbiological B class, whereas all the manufacturing process in SSCF facility is performed under a laminar air flow (class A) decreasing the contamination risk and justifying a lower number of in-process controls. This aspect is of importance as environmental controls generate significant costs and the financial sustainability of innovative cell-based therapy remains a well-known challenge [25]. Of note, experience with the SVF consistently shows that the greater contamination risk is coming from the adipose tissue harvesting and the associated break-in of the skin barrier rather than manufacturing environment. So, having clear guidelines seems crucial for that key aspect of SVF manufacturing.

SVF being a heterogeneous mixture of cells, flow cytometry analysis of the various cell subsets is an essential part of the end-product qualification but remains poorly standardized between users. The only ISCT-based recommendations dates from 2013 and has the limitation of only looking at percentages of positivity without identifying all cell subsets. Based on a common flow cytometry gating strategy ensuring comparability of results, we evidenced that the SVF composition was not exactly equivalent between the two centers. SVF obtained in SSCF facilities presented more ECs and less leukocytes and PRs. These disparities could be in line with the different production protocols. Beside the efficiency of enzymatic digestion which is dependent on adequate thermostatting, the washing step may introduce significant differences. The washing step is automated and fixed with the Celution device whereas, manually, AT can be washed as much as necessary to maximize the removal of blood cells. In addition, the well-known binding capacity of HSA [26] used to resuspend the final SVF product could nonspecifically influence the immunophenotyping analysis.

However, our work has some limitation regarding analysis of flow cytometry data. Indeed, the absence of size threshold make the interpretation difficult (supplemental figure 1), and the choice of different viability markers leads to the selection of different viable cell populations (supplemental figure 2). This underlines the urgent need to have a robust validation of analytical method according to international council for harmonization (ICH) and GMP for identification of SVF subpopulations. A collaboration between researchers, hospitals, and industries could lead to the development of lyophilized antibody mixes and dedicated kits, which should facilitate the dissemination and general approval of the technique, through the involvement of learned societies.

The deep retrospective analysis and comparison of the SVF produced by SSCF and AP-HM allowed the proposal of release acceptance criteria threshold, achievable in most cases after GMP-compliant production of SVF, whose goal is to standardize practices and upgrade the quality of the future delivered SVF. We identified four essential parameters that should be reported in any publications or researches related to SVF, and to establish the release acceptance criteria, we have chosen the best achievable thresholds in at least 80% of cases. First, the thresholds for cellular viability (≥ 80%) and yield recovery (≥ 1.50 × 10^5^ VNCs per mL) are essential to ensure an acceptable number of delivered viable cells. In 91.5% of cases, productions started with an initial volume of AT at least equal to 60 mL. Concerning the composition of the SVF, we propose thresholds for ASCs (≥ 20 %) and leukocytes (≤ 50 % ). Historically, ASCs are the cell subset of interest for the regenerative ability of AT [27]. Their implication in the biological mechanisms of SVF has been extensively studied in the context of wound healing [28, 29], angiogenesis [30–32], and scar remodeling [33, 34]. Conversely, except for a few papers reporting the involvement of macrophages in angiogenesis [35–37], the roles of leukocytes is not fully clarified. It seems therefore important to prioritize the presence of the others regenerative cells rather than blood cells. As a matter of fact, endothelial progenitor cells and pericytes have demonstrated their synergistic actions with each other and ASCs [2, 38, 39]. More recently, Kilinc et al. suggested that a 2:1 ratio of ASCs: hematopoietic cells could be a predictive factor for a successful cell therapy procedure using SVF in different conditions [40].

We identified ten articles aiming to compare SVF obtained according different manufacturing protocols [10, 13, 16, 18, 41–46]. Among them, only two articles reported the four essential parameters mentioned earlier and all of them reach the defined threshold (for Güven and coll.: more than 90% viability, 1.60 × 10^5^ VNCs per mL, 40% of ASCs and 35% of leukocytes [16]; for Condé-Green and coll., a viability between 80 and 90%, a yield recovery of 2.30 × 10^5^ VNCs per mL, 60% of ASCs and 32% of leukocytes [18]).

Finally, the proposed thresholds for the main criteria related to the final biological product (viability, recovery yield, proportions of stem cells of interest, and leukocytes contaminant) guarantee manufacturing quality. While these criteria have the advantage of being compatible whatever the production method and indication selected, they will certainly have to be refined later on. Indeed, true potency assays aiming to evaluate the functionality of the cells would be the most appropriate method to anticipate the therapeutic efficacy of a batch of FVS. Unfortunately, these tests remain complex to implement and their use is not widely spread. Conversely, the systematic biological characterization initiated in this work is easier and faster to perform in specialized manufacturing centers. From our point of view, reaching the threshold we defined for these classical parameters is the first step to the future identification of potency tests associated. We could anticipate that retrospective analysis of exhaustively characterized SVFs could reveal correlations between the proportion of a cell subset and a specific therapeutic benefit.

## Conclusions

This study identified release acceptance criteria of SVF-based therapeutic product and provides initial data that will participate to the standardization and optimization of manufacturing approaches and could bring new perspectives for cell engineering strategies.

## Supplementary Information

**Additional file 1: Supplemental Table 1.** Clinical trials of the AP-HM cohort. Detailed of the clinical trials whose patients were included in the study. ANSM : *Agence nationale de sécurité du médicament et des produits de santé*. **Supplemental Table 2.** Antibodies references for SSCF and AP-HM protocols. **Figure Supplemental 1.** Representative images of density plots with or without size scatter threshold. A: Representative density plots of a SVF from SSCF cohort acquired using a flow cytometry protocol with SS threshold and analyzed using the common gating strategy; left: selection of the NC using SS and Syto40; right, discrimination inside CD45- cell population of PR, EC, ASC on the basis of their CD146 and CD34 expression. B: Representative density plots of a SVF from SSCF cohort acquired using a flow cytometry protocol without SS threshold and analyzed using the common gating strategy; left: selection of the NC using SS and a the Syto40, nonspecific events interfered with a strict selection of NC; right: discrimination inside CD45- population of PR, EC, ASC on the basis of their CD146 and CD34 expression, EC and ASC are not clearly discriminated. SSCF: Swiss Stem Cell Foundation. EC: endothelial cell. ASC: adipose-derived stromal cell. Leuk: leukocytes. PR: pericytes. SS: size scatter. NC: nucleated cells. **Figure Supplemental 2.** Representative images of 7 AAD and DAPI staining. A: Profile of viability with the DAPI marker. B: profile of viability with the 7 AAD marker.

## Data Availability

The datasets generated and/or analyzed during the current study are not publicly available due to privacy but are available from the corresponding author on reasonable request.

## Abbreviations

AP-HM: Assistance Publique-Hôpitaux de Marseille
AT: Adipose tissue
ASC: Adipose-derived stromal cell
ATMP: Advanced therapy medicinal product
DPBS: Dulbecco’s phosphate-buffered saline
EC: Endothelial cell
GMP: Good manufacturing practices
HSA: Human serum albumin
ICH: International council for harmonization
MSC: Mesenchymal stem cell
NC: Nucleated cell
PR: Pericyte
RL: Ringer’s lactate
SD: Standard deviation
SSCF: Swiss Stem Cell Foundation
SVF: Stromal vascular fraction
VNC: Viable nucleated cell
