# Supplementary material for: Inter-center comparison of good manufacturing practices-compliant stromal vascular fraction and proposal for release acceptance criteria: a review of 364 productions
Source: Stem Cell Res Ther. 2021 Jul 1;12:373. doi: 10.1186/s13287-021-02445-z (PMC8252207; doi:10.1186/s13287-021-02445-z)
Supplement: Supplementary file 1 — Additional file 1: Supplemental Table 1. Clinical trials of the AP-HM cohort. Detailed of the clinical trials whose patients were included in the study. ANSM : Agence nationale de sécurité du médicament et des produits de santé. Supplemental Table 2. Antibodies references for SSCF and AP-HM protocols. Figure Supplemental 1. Representative images of density plots with or without size scatter threshold. A: Representative density plots of a SVF from SSCF cohort acquired using a flow cytometry protocol with SS threshold and analyzed using the common gating strategy; left: selection of the NC using SS and Syto40; right, discrimination inside CD45- cell population of PR, EC, ASC on the basis of their CD146 and CD34 expression. B: Representative density plots of a SVF from SSCF cohort acquired using a flow cytometry protocol without SS threshold and analyzed using the common gating strategy; left: selection of the NC using SS and a the Syto40, nonspecific events interfered with a strict selection of NC; right: discrimination inside CD45- population of PR, EC, ASC on the basis of their CD146 and CD34 expression, EC and ASC are not clearly discriminated. SSCF: Swiss Stem Cell Foundation. EC: endothelial cell. ASC: adipose-derived stromal cell. Leuk: leukocytes. PR: pericytes. SS: size scatter. NC: nucleated cells. Figure Supplemental 2. Representative images of 7 AAD and DAPI staining. A: Profile of viability with the DAPI marker. B: profile of viability with the 7 AAD marker. [file 13287_2021_2445_MOESM1_ESM.zip › Additional files/Additional files_Tables.docx]

**Supplemental Table 1.** Clinical trials of the AP-HM cohort. Detailed of the clinical trials whose patients were included in the study. ANSM : *Agence nationale de sécurité du médicament et des produits de santé*.

**Supplemental Table 2.** Antibodies references for SSCF and AP-HM protocols.

| Clinical trial | Clinical trial name | Authorization number by ANSM | Production period | Targeted pathology | Number of patients |
| --- | --- | --- | --- | --- | --- |
| NCT01813279 | Scleradec I | TC309 | December 2012 – April 2013 | Systemic scleroderma | 12 |
| NCT02558543 | Scleradec II | N° 1411449A-62 | October 2015 – January 2018 | Systemic scleroderma | 40 |
| NCT02520843 | Adicrohn I | N° 150303A-62 | December 2015 – March 2017 | Crohn disease | 14 |
| NCT02622464 | Cell Cord I | N° 150163A-62 | April 2016 – August 2017 | Dysphonia | 8 |

**Supplemental Table 1.**

|  | **Specificity** | **Fluorochrome** | **Supplier** | **Reference** | **Volume** |
| --- | --- | --- | --- | --- | --- |
| SSCF | CD146 | PE (Phycoérythrin) | Beckman Coulter | Duraclone Mix Lugano B38646 | Lyophilized |
|  | CD34 | AA750 (Allophycocyanin-Alexa Fluor 750) |  |  |  |
|  | CD45 | KrO (Krome Orange) |  |  |  |
|  | Syto40 | NA | Life Technologies | S11351 | 2μL (10-fold diluted) |
|  | 7-AAD | 7-aminoactinomycine D | Beckman Coulter | A07704 | 10μL |
| AP-HM | CD90 | FITC (Fluorescein isothiocyanate ) | Beckman Coulter | IM1839U | 10μL |
|  | CD146 | PE (Phycoérythrin) | Beckman Coulter | PN A07483 | 10μL |
|  | CD34 | ECD (Phycoérythrin - Texas Red-X) | Beckman Coulter | IM2709U | 10μL |
|  | CD45 | PC5 (Phycoérythrin Cyanin 5.1) | Beckman Coulter | A07785 | 10μL |
|  | DRAQ5 | 1, 5-bis{[2-(di-methylamino)ethyl]amino}-4, 8-dihydroxyanthracene-9, 10-dione | eBioscience | 65-0880-96 | 10μL (25-fold diluted) |
|  | NucBlue | DAPI (4′,6-diamidino-2-phenylindole) | Thermo Fisher Scientific | R37606 | 5μL |

**Supplemental Table 2.**
