# Supplementary figures and images for: Inter-center comparison of good manufacturing practices-compliant stromal vascular fraction and proposal for release acceptance criteria: a review of 364 productions
Source: Stem Cell Res Ther. 2021 Jul 1;12:373. doi: 10.1186/s13287-021-02445-z (PMC8252207; doi:10.1186/s13287-021-02445-z)

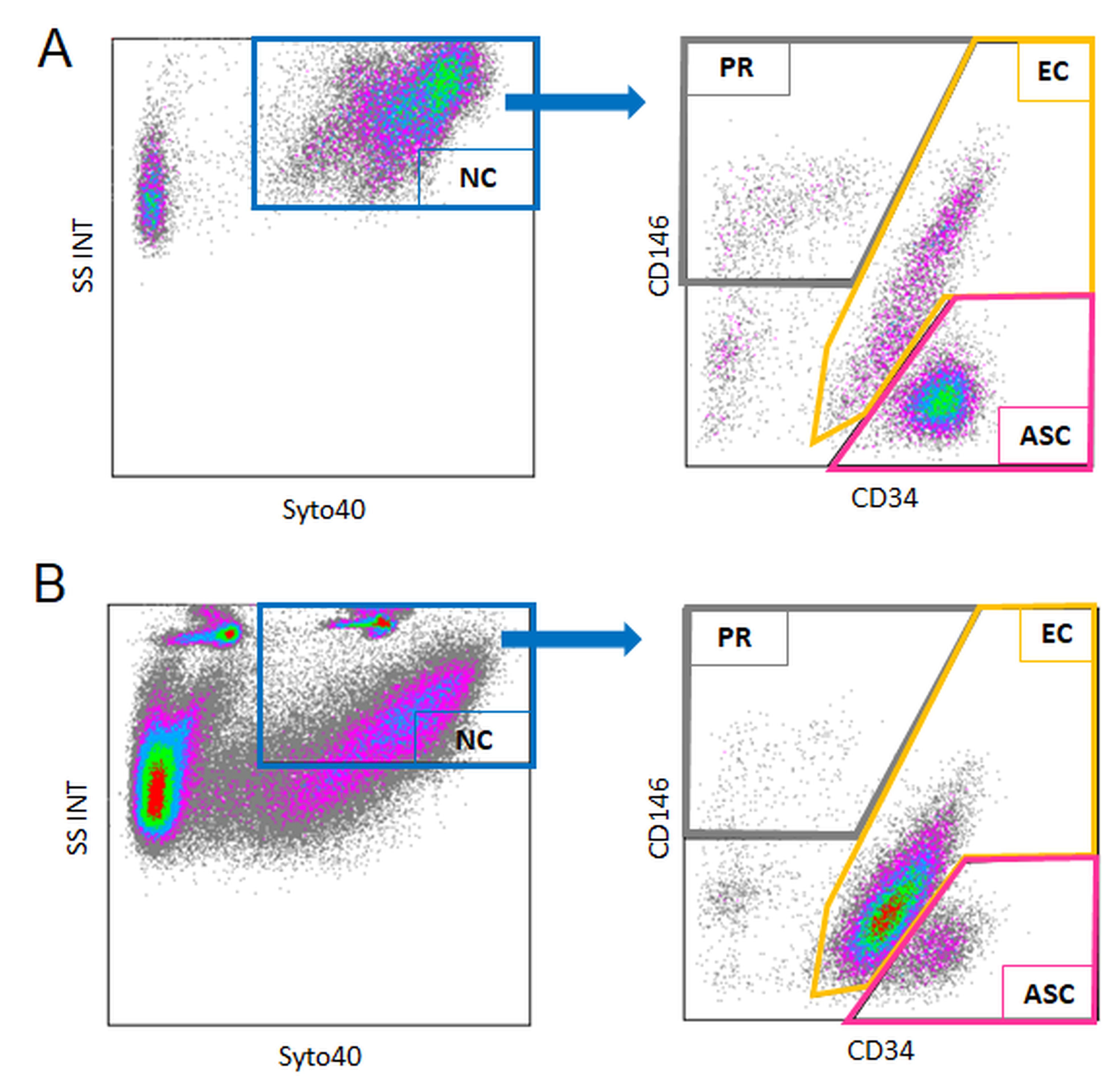

Supplement: Supplementary file 1 — Additional file 1: Supplemental Table 1. Clinical trials of the AP-HM cohort. Detailed of the clinical trials whose patients were included in the study. ANSM : Agence nationale de sécurité du médicament et des produits de santé. Supplemental Table 2. Antibodies references for SSCF and AP-HM protocols. Figure Supplemental 1. Representative images of density plots with or without size scatter threshold. A: Representative density plots of a SVF from SSCF cohort acquired using a flow cytometry protocol with SS threshold and analyzed using the common gating strategy; left: selection of the NC using SS and Syto40; right, discrimination inside CD45- cell population of PR, EC, ASC on the basis of their CD146 and CD34 expression. B: Representative density plots of a SVF from SSCF cohort acquired using a flow cytometry protocol without SS threshold and analyzed using the common gating strategy; left: selection of the NC using SS and a the Syto40, nonspecific events interfered with a strict selection of NC; right: discrimination inside CD45- population of PR, EC, ASC on the basis of their CD146 and CD34 expression, EC and ASC are not clearly discriminated. SSCF: Swiss Stem Cell Foundation. EC: endothelial cell. ASC: adipose-derived stromal cell. Leuk: leukocytes. PR: pericytes. SS: size scatter. NC: nucleated cells. Figure Supplemental 2. Representative images of 7 AAD and DAPI staining. A: Profile of viability with the DAPI marker. B: profile of viability with the 7 AAD marker. [file 13287_2021_2445_MOESM1_ESM.zip › Additional files/supplemental 1.jpg]

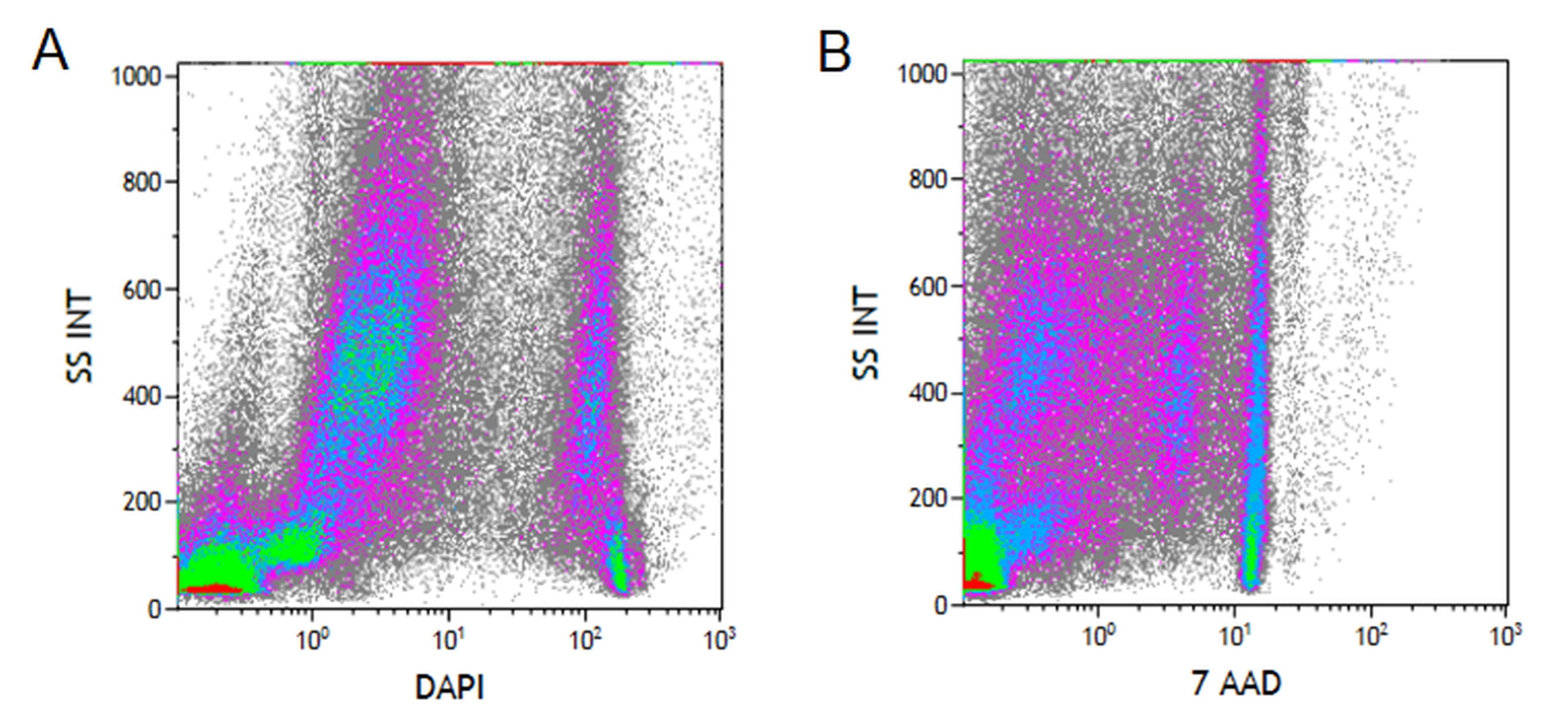

Supplement: Supplementary file 1 — Additional file 1: Supplemental Table 1. Clinical trials of the AP-HM cohort. Detailed of the clinical trials whose patients were included in the study. ANSM : Agence nationale de sécurité du médicament et des produits de santé. Supplemental Table 2. Antibodies references for SSCF and AP-HM protocols. Figure Supplemental 1. Representative images of density plots with or without size scatter threshold. A: Representative density plots of a SVF from SSCF cohort acquired using a flow cytometry protocol with SS threshold and analyzed using the common gating strategy; left: selection of the NC using SS and Syto40; right, discrimination inside CD45- cell population of PR, EC, ASC on the basis of their CD146 and CD34 expression. B: Representative density plots of a SVF from SSCF cohort acquired using a flow cytometry protocol without SS threshold and analyzed using the common gating strategy; left: selection of the NC using SS and a the Syto40, nonspecific events interfered with a strict selection of NC; right: discrimination inside CD45- population of PR, EC, ASC on the basis of their CD146 and CD34 expression, EC and ASC are not clearly discriminated. SSCF: Swiss Stem Cell Foundation. EC: endothelial cell. ASC: adipose-derived stromal cell. Leuk: leukocytes. PR: pericytes. SS: size scatter. NC: nucleated cells. Figure Supplemental 2. Representative images of 7 AAD and DAPI staining. A: Profile of viability with the DAPI marker. B: profile of viability with the 7 AAD marker. [file 13287_2021_2445_MOESM1_ESM.zip › Additional files/Supplemental 2.jpg]
